# Do-Not-Resuscitate Orders and Outcomes for Patients with Pancreatic Cancer

**DOI:** 10.1089/pancan.2022.0006

**Published:** 2022-10-06

**Authors:** Qiang Hao, Joel E. Segel, Niraj J. Gusani, Christopher S. Hollenbeak

**Affiliations:** ^1^ Department of Health Policy and Administration, Pennsylvania State University, University Park, Pennsylvania, USA.; ^2^ Penn State Cancer Institute, Hershey, Pennsylvania, USA.; ^3^ Department of Public Health Sciences, College of Medicine, The Pennsylvania State University, Hershey, Pennsylvania, USA.; ^4^ Section of Surgical Oncology, Division of Surgery, Baptist MD Anderson Cancer Center, Jacksonville, Florida, USA.; ^5^ Department of Surgery, Penn State Milton S. Hershey Medical Center, Hershey, Pennsylvania, USA.

**Keywords:** do-not-resuscitate order, pancreatic cancer, mortality, cost

## Abstract

**Background:**

The impact of the do-not-resuscitate (DNR) order on patients with pancreatic cancer remains uncertain. In this study, we evaluated whether DNR status was associated with in-hospital mortality and costs for inpatient stay among patients hospitalized with pancreatic cancer.

**Methods:**

Data were obtained from the National Inpatient Sample, Healthcare Cost and Utilization Project, which represents ∼20% of all discharges from US community hospitals; 40,246 pancreatic cancer admissions between 2011 and 2016 were included. Mortality was modeled using a logistic regression model; costs for inpatient stay were modeled using a multivariable generalized linear regression model.

**Results:**

The sample included 6041 (15%) patients with a documented DNR order. After controlling for covariates, patients with a DNR order had approximately six times greater odds of mortality compared with patients without a DNR order (odds ratio 5.90, *p* < 0.0001). Compared with patients who survived without a DNR order during the hospital stay, patients who had a DNR order and died during the hospital stay had significantly lower costs (−US$983; *p* = 0.0270), and patients who died without a DNR order during the hospital stay had significantly higher costs (US$5638; *p* < 0.0001). Patients who survived with a DNR order had costs that were not significantly different from patients who survived without a DNR order.

**Conclusions:**

The presence of a DNR order among patients with pancreatic cancer was significantly associated with higher mortality risk as well as lower costs for patients who died during the hospital stay. However, DNR status was not significantly associated with costs for pancreatic cancer patients who were discharged alive.

## Introduction

Documenting end-of-life (EOL) care preferences is important for patients with serious illnesses. Engaging in advance care planning—having discussions with family, caregivers, and clinicians about their preferences for EOL care—has been shown to improve the quality of life for patients and their families.^1–3^ Do-not-resuscitate (DNR) orders provide a mechanism to preserve patient autonomy by documenting a preference to withhold cardiopulmonary resuscitation (CPR) in the event of cardiopulmonary arrest and can be an essential part of an advance care plan.^3–7^ Importantly, a DNR order has no effect on any curative treatment besides CPR.^5,6^

Some previous studies have reported that DNR orders are associated with higher mortality, but better reported quality of life.^7–10^ Less was known about trends in the use of DNR orders and the relationship between DNR orders and outcomes among patients with high mortality conditions such as pancreatic cancer, which is the third leading cause of cancer mortality in the United States in 2022.^11–15^

According to the American Cancer Society, the combined overall 5-year relative survival rate for all stages of pancreatic cancer is ∼11%.^15^ Yet, almost half of these patients are not diagnosed until late in the course of the illness, which leads to a worse prognosis and a 5-year relative survival rate of only 4%.^16–20^ Therefore, DNR orders may be especially important for patients with a pancreatic cancer diagnosis. Even though previous studies have shown that DNR orders appear to lower hospitalization costs in the last week of life among patients with advanced cancer, there may be differences in cost savings across cancer sites.^9,21,22^

Furthermore, high health care costs accompany advanced cancer throughout the entire inpatient stay. Thus, total hospitalization costs of an inpatient stay potentially provide a better understanding of the association with DNR orders than costs in just the last week of life.

Our primary objective was to use national data to examine whether DNR status has a significant association with in-hospital mortality and costs for the inpatient stay among hospitalized patients with pancreatic cancer. In this study, we estimate trends in DNR order utilization among patients with pancreatic cancer, and we report the characteristics of patients with a DNR order as well as their mortality and costs compared with those of patients without a DNR order.

We hypothesized that the proportion of patients with a DNR order would increase annually and that DNR orders among patients with pancreatic cancer would be associated with significantly higher mortality risk, together with significantly lower costs for the inpatient stay for the patients who died during the hospital stay.

## Methods

### Data sources

Data for this study were obtained from the 2011 to 2016 National Inpatient Sample (NIS), Healthcare Cost and Utilization Project (HCUP), Agency for Healthcare Research and Quality (AHRQ).^23^ The data set, which includes >1000 hospitals, approximates a 20% stratified sample of all discharges from US community hospitals and is the largest, publicly available, all-payer, inpatient health care database in the United States.^23^

### Cohort

This study examined a cohort of patients hospitalized with a principal diagnosis of pancreatic cancer. Pancreatic cancer was identified using the principal International Classification of Diseases, 9th or 10th Revision, Clinical Modification (ICD-9-CM or ICD-10-CM) diagnosis codes for pancreatic cancer (ICD-9-CM: 157.X; and ICD-10-CM: C25.X). All four stages of pancreatic cancer were included. Initially, 44,268 patients with pancreatic cancer admitted between 2011 and 2016 in the NIS data set were identified.

**FIG. 1.**
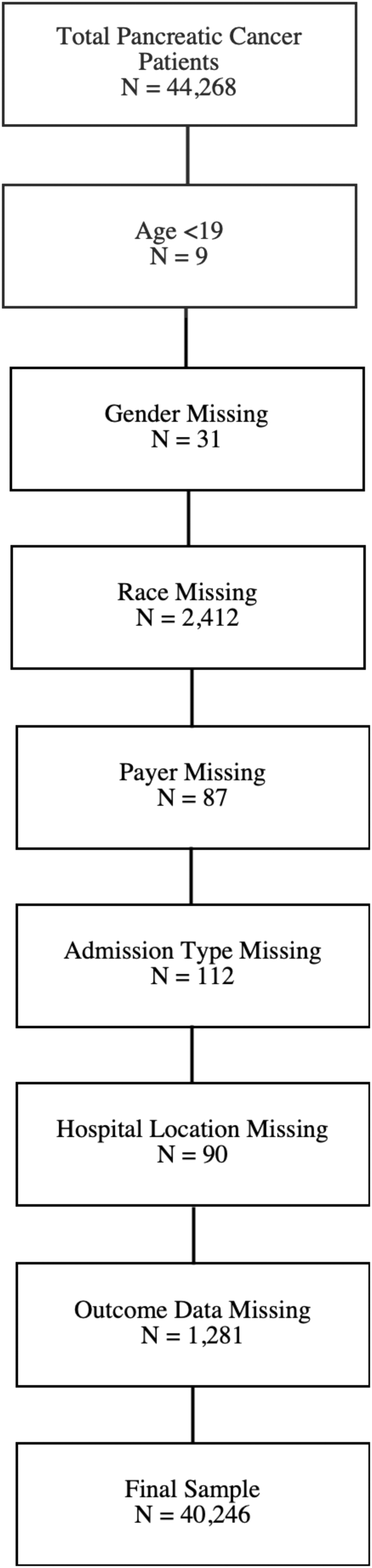
Determination of the study cohort.

This study focused on adults and excluded 4022 patients with missing covariates. After all exclusion criteria were applied, the final study sample included 40,246 patients, and 22.71% received pancreatic surgery (Fig. 1).

### Outcomes

This study examined two outcomes: in-hospital mortality and costs. Mortality was defined as death during hospitalization before discharge. Costs represented the total hospitalization costs for the inpatient stay from admission to discharge or death. Costs were estimated using a hospital-specific cost-to-charge ratio methodology that estimated costs as a percentage of hospital charges and the sum of all departmental costs.^24^

In addition, costs were inflated to 2018 US dollars using the medical care component of the Consumer Price Index.^25^

### Covariates

The primary covariate of interest was an indicator of whether the patient had a DNR order. DNR order was identified using ICD-9-CM code V49.86 or ICD-10-CM code Z66.^26,27^

We controlled for several other covariates in the study, including demographic characteristics (age, sex, and race/ethnicity), the Charlson comorbidity index (CCI) (score of 0–2, 3–5, 6–8, and ≥9), location of tumor (head, body, tail, other specified sites, and part unspecified), primary payer (Medicare, Medicaid, commercial, and other), admission type (elective or nonelective), teaching hospital status (rural nonteaching, urban nonteaching, and urban teaching), region of the country (Northeast, Midwest, South, and West), an indicator for surgery during the admission, and indicators for year of admission (2011 to 2016) to control for other secular trends.

### Statistical analyses

The statistical analysis was designed to determine whether there was a significant association between DNR status and in-hospital mortality or in-hospital costs among patients diagnosed with pancreatic cancer. Comparisons of demographics and other patient and disease characteristics were made between patients with and without a DNR order using t-tests for continuous variables and chi-squared tests for categorical variables.

A logistic regression model was used to estimate the association between DNR status and in-hospital mortality, after controlling for all the aforementioned covariates. Odds ratios (ORs) were reported from the logistic regression model. Costs were modeled using a multivariable generalized linear regression model that assumed a gamma family of distributions and a log link function.^28,29^

This model was chosen to account for skewness in cost data following the health care cost literature. All statistical analyses were performed using Stata (version 16; StataCorp LLP, College Station, TX). Statistical significance for all analyses was defined by *p*-values <0.05.

## Results

### Descriptive statistics

Of the 40,246 hospitalized pancreatic cancer patients included in this study, 6041 (15%) had a DNR order. Patients with a DNR order had an unadjusted 25.2% in-hospital mortality versus 4.5% for patients without a DNR order. The mean unadjusted costs were $15,217 and $22,622, respectively, for patients with and without a DNR order.

Patient characteristics stratified by DNR status are shown in Table 1. Across all factors, we found significant differences between patients with a DNR order and those without. Patients with a DNR order were significantly older than patients without a DNR order (72.5 years vs. 67.4 years, *p* < 0.0001) and more likely to be female (52.7% vs. 49.3%, *p* < 0.0001). Patients with a DNR order had a higher comorbidity burden, as measured by a higher CCI score (7.27 vs. 6.04, *p* < 0.0001).

**Table 1.** Characteristics of Pancreatic Cancer Patients, by Do-Not-Resuscitate Order Status

Variable	DNR order			No DNR order			
	Total	Survived	Died	Total	Survived	Died	
	(***N*** = 6041)	(***N*** = 4519)	(***N*** = 1522)	(***N*** = 34,205)	(***N*** = 32,657)	(***N*** = 1548)	***p***
Age, years	72.5	73.6	69.2	67.4	67.3	68.7	<0.0001
19–59	16.5%	14.5%	22.5%	25.6%	25.7%	22.9%	
60–69	23.0%	21.4%	27.8%	30.5%	30.6%	28.0%	
70–79	27.0%	27.4%	26.0%	27.1%	27.0%	28.9%	
≥80	33.5%	36.8%	23.7%	16.8%	16.6%	20.3%	
Sex							
Female	52.7%	55.1%	45.3%	49.3%	49.6%	44.0%	<0.0001
Male	47.3%	44.9%	54.7%	50.7%	50.4%	56.0%	
Race/ethnicity							
White non-Hispanic	72.8%	74.2%	68.3%	72.0%	72.0%	71.4%	0.2400
Black non-Hispanic	13.4%	12.7%	15.6%	13.5%	13.5%	14.1%	
Hispanic	7.5%	7.2%	8.3%	8.2%	8.2%	7.9%	
Asian	3.5%	3.4%	3.9%	3.2%	3.2%	3.4%	
Other	2.8%	2.5%	3.9%	3.1%	3.1%	3.2%	
CCI score	7.27	7.20	7.47	6.04	6.00	7.04	
0–2	11.4%	11.3%	11.7%	20.0%	20.2%	14.8%	<0.0001
3–5	20.5%	21.6%	17.1%	28.0%	28.4%	20.1%	
6–8	26.5%	25.8%	28.6%	23.0%	22.9%	26.6%	
≥9	41.6%	41.2%	42.5%	29.0%	28.5%	38.6%	
Location of tumor							
Head	27.9%	31.0%	18.5%	45.3%	46.4%	22.8%	<0.0001
Body	4.6%	4.9%	3.8%	6.8%	6.9%	3.9%	
Tail	7.4%	7.9%	6.2%	8.1%	8.2%	6.4%	
Other specified sites	7.7%	7.5%	8.4%	9.7%	9.7%	8.7%	
Part unspecified	53.7%	50.1%	64.2%	31.7%	30.4%	59.4%	
Payer							
Medicare	64.3%	70.0%	47.3%	56.3%	56.6%	48.8%	<0.0001
Medicaid	7.0%	6.5%	8.4%	8.7%	8.8%	6.6%	
Commercial	21.2%	18.2%	29.9%	29.0%	28.8%	32.9%	
Other	7.6%	5.3%	14.4%	6.1%	5.8%	11.6%	
Admission type							
Elective	13.8%	10.4%	23.7%	36.4%	36.8%	28.7%	<0.0001
Nonelective	86.2%	89.6%	76.3%	63.6%	63.2%	71.3%	
Teaching hospital							
Rural	6.4%	5.6%	8.8%	4.5%	4.1%	13.6%	<0.0001
Urban nonteaching	28.3%	27.9%	29.4%	23.5%	23.2%	30.5%	
Urban teaching	65.3%	66.5%	61.8%	72.0%	72.7%	55.9%	
Region							
Northeast	22.5%	21.6%	25.0%	21.9%	21.9%	22.7%	<0.0001
Midwest	20.0%	20.6%	18.5%	19.7%	19.7%	19.7%	
South	37.4%	37.1%	38.6%	41.4%	41.3%	43.3%	
West	20.0%	20.7%	17.9%	17.0%	17.1%	14.2%	
Surgery							
No	98.2%	98.7%	96.6%	73.6%	72.9%	88.2%	<0.0001
Yes	1.8%	1.3%	3.4%	26.4%	27.1%	11.8%	

CCI, Charlson comorbidity index; DNR, do-not-resuscitate.

They were also more likely to have Medicare as the primary payer (64.3% vs. 56.3%, *p* < 0.0001) and more likely to be treated in a rural nonteaching hospital (6.4% vs. 4.5%, *p* < 0.0001) or urban nonteaching hospital (28.3% vs. 23.5%, *p* < 0.0001). We further included stratification by four groups representing DNR and survival status, as shown in Table 1. In the DNR group, patients who died in the hospital were more likely to be male (45.3% female vs. 55.1% male) and have fewer tumors at the head of the pancreas (18.5% vs. 31.0%) and were less likely to have Medicare as the primary payer (47.3% vs. 70.0%), compared with patients who survived.

Among patients without a DNR order, those who died were less likely to have tumors at the head of the pancreas (22.8% vs. 46.4%) and were more likely to have been treated in a rural nonteaching hospital (13.6% vs. 4.1%), compared with patients who survived. Patients who had surgery were less likely to have a DNR order (1.8% vs. 26.4%) compared with patients who had no surgery.

**FIG. 2.**
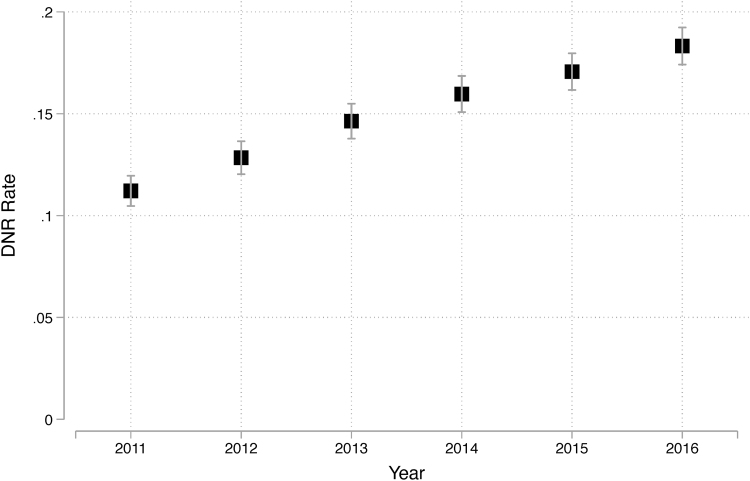
Proportion of patients with pancreatic cancer with a DNR order, stratified by year of admission. DNR, do-not-resuscitate.

Notably, the proportion of patients with a DNR order increased annually from 12.9% in 2011 to 21.1% in 2016 (*p* < 0.0001), as shown in Figure 2. This increase in DNR order utilization may be due to several factors, including increasing use by billing coders, more effective communication about EOL care between physicians and patients, and more effective advance care planning on the part of patients.

### Association between DNR order and mortality

The results of the logistic regression model for in-hospital mortality are presented in Table 2. After controlling for all covariates, patients with a DNR order had approximately six times greater odds of mortality compared with patients without a DNR order (OR 5.90, confidence interval [95% CI] 5.41–6.42, *p* < 0.0001). In addition, several other patient characteristics were associated with mortality based on the logistic regression model with all covariates included. Women had a significantly lower risk of mortality compared with men (OR 0.79, 95% CI 0.73–0.86, *p* < 0.0001).

**Table 2.** Results of a Logistic Regression Model of the Effect of the Do-Not-Resuscitate Order on Mortality, Controlling for Other Covariates

Variable	Odds ratio	95% Confidence interval		***p***
		Lower	Upper	
DNR	5.90	5.41	6.42	<0.0001
Age, years				
19–59	Reference			
60–69	1.23	1.09	1.38	0.0010
70–79	1.57	1.37	1.79	<0.0001
≥80	1.45	1.25	1.67	<0.0001
Sex				
Male	Reference			
Female	0.79	0.73	0.85	<0.0001
Race/ethnicity				
White non-Hispanic	Reference			
Black non-Hispanic	1.24	1.10	1.39	<0.0001
Hispanic	1.10	0.95	1.28	0.2000
Asian	1.24	1.00	1.55	0.0510
Other	1.30	1.04	1.62	0.0210
CCI score				
0–2	Reference			
3–5	1.01	0.88	1.16	0.8660
6–8	1.35	1.18	1.54	<0.0001
≥9	1.46	1.29	1.66	<0.0001
Location of tumor				
Head	Reference			
Body	1.09	0.89	1.34	0.3890
Tail	1.27	1.07	1.51	0.0060
Other specified sites	1.63	1.40	1.90	<0.0001
Part unspecified	2.54	2.30	2.80	<0.0001
Payer				
Medicare	Reference			
Medicaid	1.45	1.22	1.72	<0.0001
Commercial	1.93	1.73	2.15	<0.0001
Other	2.99	2.58	3.45	<0.0001
Admission type				
Elective	Reference			
Nonelective	0.71	0.64	0.78	<0.0001
Teaching hospital				
Urban teaching	Reference			
Rural	2.38	2.06	2.74	<0.0001
Urban nonteaching	1.27	1.16	1.39	<0.0001
Region				
Northeast	Reference			
Midwest	0.76	0.67	0.86	<0.0001
South	0.77	0.69	0.86	<0.0001
West	0.71	0.62	0.81	<0.0001
Surgery				
No	Reference			
Yes	0.57	0.48	0.67	<0.0001
Year				
2011	Reference			
2012	0.87	0.76	0.99	0.0360
2013	0.87	0.76	1.00	0.0430
2014	0.80	0.70	0.92	0.0010
2015	0.69	0.60	0.79	<0.0001
2016	0.66	0.58	0.76	<0.0001

Patients who were non-Hispanic Black had significantly higher odds of mortality compared with non-Hispanic White patients (OR 1.24, 95% CI 1.10–1.39, *p* < 0.0001). Additionally, patients who had a CCI of 6–8 or ≥9 had significantly greater risk of mortality (OR 1.35, 95% CI 1.18–1.54, *p* < 0.0001, and OR 1.46, 95% CI 1.29–1.66, *p* < 0.0001, respectively). Patients with tumors of the tail and other sites of the pancreas had significantly higher odds of mortality compared with tumors of the head of the pancreas (OR 1.27, 95% CI 1.07–1.51, *p* = 0.0060, and OR 1.63, 95% CI 1.40–1.90, *p* < 0.0001, respectively).

Medicare was associated with a significantly lower risk of mortality (*p* < 0.0001). Patients treated at rural (OR 2.38, 95% CI 2.06–2.74, *p* < 0.0001) and urban nonteaching hospitals (OR 1.27, 95% CI 1.16–1.39, *p* < 0.0001) had significantly higher risk of mortality than patients treated at urban teaching hospitals.

### Association between DNR order and costs for inpatient stay

Results from the generalized linear model estimates of costs are presented in Table 3. The model captured the interaction between DNR orders and mortality and showed that patients with a DNR order who died in the hospital had significantly lower costs (−US$983; 95% CI −1855 to −111; *p* = 0.0270) compared with patients with no DNR order who survived.

**Table 3.** Results of a Generalized Linear Model of the Effect of the Do-Not-Resuscitate Order on Costs, Controlling for Other Covariates

Variable	Marginal effect	95% Confidence interval		***p***
		Lower	Upper	
No DNR order/survived	Reference			
No DNR order/died	$5638	$4479	$6797	<0.0001
DNR order/survived	−$456	−$1017	$104	0.1100
DNR order/died	−$983	−$1855	−$111	0.0270
Age, years				
19–59	Reference			
60–69	−$528	−$1030	−$27	0.0390
70–79	−$1178	−$1766	−$589	<0.0001
≥80	−$2795	−$3399	−$2191	<0.0001
Sex				
Male	Reference			
Female	−$622	−$970	−$274	<0.0001
Race/ethnicity				
White non-Hispanic	Reference			
Black non-Hispanic	$1887	$1322	$2451	<0.0001
Hispanic	$954	$265	$1643	0.0070
Asian	$2120	$1016	$3225	<0.0001
Other	$2444	$1314	$3574	<0.0001
CCI score				
0–2	Reference			
3–5	$2428	$1873	$2983	<0.0001
6–8	$2545	$1962	$3128	<0.0001
≥9	$4231	$3660	$4801	<0.0001
Location of tumor				
Head	Reference			
Body	−$2967	−$3589	−$2344	<0.0001
Tail	−$4099	−$4643	−$3556	<0.0001
Other specified sites	−$643	−$1246	−$39	0.0370
Part unspecified	−$5527	−$5907	−$5147	<0.0001
Payer				
Medicare	Reference			
Medicaid	$93	−$648	$834	0.8050
Commercial	−$1234	−$1717	−$750	<0.0001
Other	−$2942	−$3623	−$2262	<0.0001
Admission type				
Elective	Reference			
Nonelective	−$88	−$542	$365	0.7030
Teaching hospital				
Urban teaching	Reference			
Rural	−$5468	−$6082	−$4855	<0.0001
Urban nonteaching	−$2719	−$3117	−$2322	<0.0001
Region				
Northeast	Reference			
Midwest	−$2411	−$2909	−$1912	<0.0001
South	−$2231	−$2687	−$1775	<0.0001
West	$3981	$3324	$4638	<0.0001
Surgery				
No	Reference			
Yes	$23,874	$22,926	$24,822	<0.0001
Year				
2011	Reference			
2012	−$946	−$1521	−$370	0.0010
2013	−$1278	−$1848	−$707	<0.0001
2014	−$1565	−$2131	−$1000	<0.0001
2015	−$1890	−$2447	−$1333	<0.0001
2016	−$2648	−$3187	−$2108	<0.0001

Conversely, compared with patients who survived with no DNR order, patients who died without a DNR order had significantly higher costs (US$5638; 95% CI 4479–6797; *p* < 0.0001). Patients who survived with a DNR order had costs that were not significantly different from patients who survived without a DNR order. There were several other covariates that were associated with costs. Patients over 60 years of age had significantly lower costs relative to those of younger age, and this difference increased with age. Women had significantly lower costs relative to men (−US$622; 95% CI −970 to −274; *p* < 0.0001).

Race/ethnicity was significantly associated with costs, with all racial/ethnic groups experiencing higher costs relative to non-Hispanic White patients, including non-Hispanic Black patients (US$1187; 95% CI 1322–2451; *p* < 0.0001), Hispanic patients (US$954; 95% CI 265–1643; *p* = 0.0070), Asian patients (US$2120; 95% CI 1016–3225; *p* < 0.0001), and other race/ethnicity (US$2444; 95% CI 1314–3574; *p* < 0.0001) groups.

Location of the tumor was significantly associated with costs; patients with a tumor in the body or tail of the pancreas had significantly lower costs (US$2967 and US$4099, respectively) compared with patients with tumors of the head of the pancreas, both *p* < 0.0001. Commercially insured patients had lower costs than Medicare patients (−US$1234; 95% CI −1717 to −750; *p* < 0.0001). Patients who underwent a surgical resection during admission had significantly higher costs of US$23,874 compared with patients who did not undergo surgery (95% CI 22,926–24,822, *p* < 0.0001).

## Discussion

This study showed that between 2011 and 2016, ∼15% of patients with pancreatic cancer had a DNR order documented with an ICD-9 or ICD-10 diagnosis code in the discharge data and the proportion of patients with a DNR order grew steadily from 2011 to 2016. We also showed that the presence of a DNR order among patients with pancreatic cancer was significantly associated with higher in-hospital mortality risk as well as lower costs for the inpatient stay for the patients who died during the hospital stay.

However, DNR status was not significantly associated with costs for patients with pancreatic cancer who were discharged alive. Importantly, we provide the first estimates using nationally representative data, which include the recent sharp increase in use of DNR orders.

To our knowledge, this is the first observational study using national, administrative discharge data to evaluate the association between DNR orders and outcomes among patients with pancreatic cancer. In our study, patients with a DNR order had six times higher odds of mortality relative to those who did not have a documented DNR order. This finding is consistent with previous studies, which have shown that risk of mortality for patients with a DNR order was higher than for patients without a DNR order.^3,7,19,30,31^

Hanson et al performed a consecutive prospective cohort study on patients with stage IV cancer and concluded that patients with a DNR order had a four times higher risk of mortality than patients without a DNR order in a single-site study.^19^ In addition, Walsh et al conducted a retrospective analysis and found that patients with a DNR order had 2.5 times greater odds of incidence of postoperative mortality compared with patients without a DNR order, although data were restricted to surgeries and older data that do not capture the large increase in DNR orders in the latter parts of our data.^7^

Furthermore, a retrospective review performed by Marcia et al reported that DNR orders were associated with higher than nine times mortality among advanced cancer patients using single-site data.^3^

We also found that pancreatic cancer patients who died with a DNR order had significantly lower costs for the inpatient stay (US$983), compared with patients who did not have a DNR order and survived to discharge. There are very few studies that have measured the association between DNR orders and costs.^10^

Maksoud et al performed a retrospective chart review that ascertained the rates of DNR orders and the associated costs, which yielded results similar to ours, although their results are from nearly 30 years ago.^10^ They found that patients with a DNR order obtained in the hospital have significantly lower total hospital charges than patients without a DNR order.^10^

Recent studies were more likely to report costs of care in the week or the month before death as the main outcome to examine the association between DNR orders and costs.^9,21,22,32^ A comprehensive evaluation conducted by Garrido et al had estimated health care costs among advanced cancer patients and they did not find a significant difference in the association between DNR status and costs of care received in the week before death.^9^ However, it was a relatively small single-site study and their conclusion may have limited generalizability.^9^

Patel et al conducted a retrospective analysis in a single Veterans Affairs health care system site among patients with stage III or IV or recurrent cancer and found that advanced cancer patients who died with a stated EOL preference document had significantly lower total health care costs within 30 days of death, compared with patients who died without a stated EOL preference document.^21,22^ However, this difference was not significant if the window was extended to total health care costs within 15 months.^21,22^

The strength of our finding of cost savings associated with DNR orders in pancreatic cancer may, in part, be explained by the relatively late stage at diagnosis for many patients, the fact that surgery is often not curative, and the general poor prognosis for these patients relative to other types of advanced cancers.^33^ Therefore, a DNR effect may be more pronounced in our population relative to earlier studies that focused on other cancer sites.

Finally, the “failure to rescue” phenomenon has been previously discussed, which suggests that a DNR order may have negative effects on other curative treatments.^5^ Our analysis confirmed that patients who had a DNR order and survived to discharge had no significant differences in costs compared with patients who did not have a DNR order and survived to discharge. A study by Brovman et al also provided evidence that there was no significant difference in the incidence of 30-day complications between patients with and without a DNR order.^5^

This study has a variety of limitations. First, the tumor stage was not available in the data set, therefore we could not restrict to only patients with the most advanced disease or control for the effect of stage on DNR order utilization or outcomes.

Second, our measure of costs included only one inpatient stay. This may underestimate all cost savings that may be potentially attributable to DNR orders because costs of patients with hospital transitions or multiple visits cannot be fully estimated using this data set. In addition, the costs of outpatient visits were not included, although we expect inpatient costs to outweigh outpatient costs.

Third, there may be other unmeasured confounders not available in the data set that could partially explain the differences in the association between DNR orders and costs, such as education level and income.

Fourth, our DNR orders were measured by the ICD-9 or ICD-10 diagnosis code, which might not fully capture the range of patient and family preferences, as well as other types of DNR orders (on admission or postadmission). However, the presence of a DNR order in the medical record suggested that it is recorded and therefore providers are likely aware of it.

Despite these limitations, our study fills important gaps in understanding the use of DNR orders among patients with pancreatic cancer. The total costs in this study reflect the value of the resources used by health care providers. Therefore, the data set allows for a reasonable way to examine the association between DNR orders and costs among pancreatic cancer patients. No previous study that we could find estimated this association in pancreatic cancer using national data.

## Conclusions

This study demonstrates that among patients hospitalized with pancreatic cancer, a DNR order was associated with higher mortality and lower costs, but DNR status had no significant association with costs for patients who survived to discharge. The primary benefit of a DNR order is that it ensures that patients receive the care they prefer. Our results show that for patients who elect for a DNR order, there is a secondary indirect benefit of reduced resource utilization.

Results of this study should inform policymakers, administrators, and health care providers as they consider guidelines for advance care planning discussions, including discussions about DNR orders, with pancreatic cancer patients.

## Data Availability

The data that support the findings of this study are available from the 2011 to 2016 National Inpatient Sample (NIS), Healthcare Cost and Utilization Project (HCUP), Agency for Healthcare Research and Quality (AHRQ).

## Abbreviations Used

AHRQ: Agency for Healthcare Research and Quality
CCI: Charlson comorbidity index
CI: confidence interval
CPR: cardiopulmonary resuscitation
DNR: do-not-resuscitate
EOL: end-of-life
HCUP: Healthcare Cost and Utilization Project
OR: odds ratio
